# Gray matter network reorganization in multiple sclerosis from 7‐Tesla and 3‐Tesla MRI data

**DOI:** 10.1002/acn3.51029

**Published:** 2020-04-07

**Authors:** Gabriel Gonzalez‐Escamilla, Dumitru Ciolac, Silvia De Santis, Angela Radetz, Vinzenz Fleischer, Amgad Droby, Alard Roebroeck, Sven G. Meuth, Muthuraman Muthuraman, Sergiu Groppa

**Affiliations:** ^1^ Department of Neurology Focus Program Translational Neuroscience (FTN) Rhine‐Main Neuroscience Network (rmn^2^) University Medical Center of the Johannes Gutenberg University Mainz Mainz Germany; ^2^ Instituto de Neurociencias de Alicante Alicante Spain; ^3^ Department of Cognitive Neuroscience Faculty of Psychology & Neuroscience Maastricht University Maastricht Netherlands; ^4^ Department of Neurology with Institute of Translational Neurology University of Münster Münster Germany

## Abstract

**Objective:**

The objective of this study was to determine the ability of 7T‐MRI for characterizing brain tissue integrity in early relapsing‐remitting MS patients compared to conventional 3T‐MRI and to investigate whether 7T‐MRI improves the performance for detecting cortical gray matter neurodegeneration and its associated network reorganization dynamics.

**Methods:**

Seven early relapsing‐remitting MS patients and seven healthy individuals received MRI at 7T and 3T, whereas 30 and 40 healthy controls underwent separate 3T‐ and 7T‐MRI sessions, respectively. Surface‐based cortical thickness (CT) and gray‐to‐white contrast (GWc) measures were used to model morphometric networks, analyzed with graph theory by means of modularity, clustering coefficient, path length, and small‐worldness.

**Results:**

7T‐MRI had lower CT and higher GWc compared to 3T‐MRI in MS. CT and GWc measures robustly differentiated MS from controls at 3T‐MRI. 7T‐ and 3T‐MRI showed high regional correspondence for CT (*r* = 0.72, *P* = 2e‐78) and GWc (*r* = 0.83, *P* = 5.5e‐121) in MS patients. MS CT and GWc morphometric networks at 7T‐MRI showed higher modularity, clustering coefficient, and small‐worldness than 3T, also compared to controls.

**Interpretation:**

7T‐MRI allows to more precisely quantify morphometric alterations across the cortical mantle and captures more sensitively MS‐related network reorganization. Our findings open new avenues to design more accurate studies quantifying brain tissue loss and test treatment effects on tissue repair.

## Introduction

Altered cortical morphology is evident from the earliest stages of multiple sclerosis (MS); thus, the characterization of cortical gray matter (GM) damage is crucial for understanding autoimmune and neurodegenerative pathology in MS and for tracking disease courses.^1^, ^2^ Non‐invasive, in vivo measures of GM integrity, through structural magnetic resonance imaging (MRI), has shed new light on the role of cortical GM during disease courses or the transition from inflammatory to degenerative stages in MS. As the extent of cortical pathology is extremely relevant for disease‐related outcomes,^3^ accurate MRI segmentation of the cerebral tissues is critical for studies evaluating therapeutic responses and disease progression. Therefore, the use of MRI at higher magnetic fields becomes highly attractive. As a result, the number of clinical and basic research studies in MS implementing ultra‐high field MRI (UHF‐MRI; i.e., ≥ 7T) has increased in recent years,^4^, ^5^, ^6^ permitting a step forward in providing a more accurate morphological depiction of subtle variations in cortical GM microstructural pathology in vivo^7^, ^8^ and increased detection of cortical lesions^9^, ^10^ compared to conventional magnetic field (1.5T and 3T) MRI. Despite the appreciable value of UHF‐MRI for the characterization of MS‐induced tissue alterations and diagnostic workup, its use still encounters technical and biological constraints,^4^ while precise estimation of cortical GM features is challenged by current analysis pipelines based on lower magnetic fields.^11^

Compared to conventional field strengths, UHF‐MRI enables an acquisition of images with higher signal‐to‐noise ratio (SNR)^12^ and tissue contrast‐to‐noise ratio (CNR)^13^, with no or only small increases in acquisition duration. Increased SNR and CNR result in data with higher spatial resolution and improved visualization of fine contrast differences,^14^ leading to reduced partial volume effects and clearer tissue boundary differentiation.^11^ The latter attribute is key for the precise estimation of cortical thickness (CT), a common and reliable measure of GM tissue loss in studies tracking neurodegeneration in MS.^15^, ^16^, ^17^, ^18^ Regional variations in tissue integrity are further captured by GM/white matter (WM) contrast (GWc), a measure of tissue‐contrast intensity differences between the boundaries of GM and WM. This morphometric measure enables the quantification of early neuronal injury and microstructural tissue alterations caused by degeneration of neural tissue.^19^

Beyond the threshold of detectability of persistent tissue loss and restoration attainable by conventional and UHF‐MRI systems, remodeling of cortical GM networks is a crucial feature of evolving MS pathology that mirrors disease‐related reorganization processes.^20^ GM network remodeling in patients with MS is imprinted as patterns of increased modularity, increased local clustering, and long‐range disconnection.^21^, ^22^ Yet, scarce studies with both 3T‐ and 7T‐MRI on the same MS patients are currently available; therefore, consistent data on the possible advances of network reconstruction based on UHF‐MRI are lacking.

In this work, we sought to address several key questions: (1) can 7T‐MRI surpass 3T‐MRI methodological constrains for characterizing the properties of GM/WM tissue boundaries? (2) how 7T‐MRI impacts the quantification of cortical morphometry in MS and with respect to healthy controls? and (3) what is the additive value of 7T‐MRI for network reconstructions from cortical GM measures in comparison to the state‐of‐the‐art 3T‐MRI? Specifically, we estimated the CT and GWc from 7T‐ and 3T‐MRI datasets obtained from MS patients and computed network metrics by means of graph theory.

## Methods

### Participants

Seven relapsing‐remitting MS (RRMS) patients (mean age ± standard deviation [SD]—41.2 ± 15.5 years, range 20–59 years, five male; Table 1) with a mean disease duration of 3.3 ± 2.9 years and seven healthy control participants (HC; mean age 39.1 ± 14.6 years, range 19–58 years; three male) were included. The level of clinical disability was measured with Expanded Disability Status Scale (EDSS). All MS patients participated in a 3T‐ and a 7T‐MRI session no more than 48 h apart. At the time of scanning, three patients were receiving disease‐modifying treatment with interferon beta‐1a, two patients with natalizumab, one patient with mitoxantrone, and one patient with glatiramer acetate. None of the patients presented neuropsychiatric comorbidities (anxiety/depression according to Hamilton depression and anxiety scale).

**Table 1:** Participants’ demographic and clinical characteristics.

Cohort	MS (3T & 7T)	HC (3T & 7T)	HC (3T only)	HC (7T only)
Mean age (± SD), years	40.4 ± 15.5	39.1 ± 14.6	29.2 ± 7.3	30.4 ± 12.1
Female/male ratio	5/2	4/3	14/16	19/21
Mean disease duration (± SD), years	3.3 ± 2.9	–	–	–
Median EDSS (range)	1.0 (0–3.5)	–	–	–
Mean WML 3T (± SD)	4.2 ± 5.6*	1.3 ± 0.5	1 ± 0.5	–
Mean WML 7T (± SD)	4.7 ± 3.2*	3.5 ± 4.9	–	1.1 ± 0.6
mean BPV 3T (± SD)	997864.7 ± 64028.9*	1054107 ± 162264.1	1045867 ± 96468.9	–
mean BPV 7T (± SD)	949882.3 ± 68526.9*	1045119.4 ± 142164	–	1018672.5 ± 86579.9

Chi‐square test compare was used to compare categorical variables (i.e., gender) across groups. ANOVA was used to compare continuous variables across groups. Asterisks indicate statistically significant values in MS when compared to those in HC. No differences in age and gender distributions were attested.

MS, multiple sclerosis; HC, healthy controls; EDSS, Expanded Disability Status Scale; WML, white matter lesions in ml; BPV, brain parenchymal volume in mm^3^.

Additionally, thirty HC (mean age 29.2 ± 7.3 years, range 20–44 years; 16 male) imaged on the same 3T MRI scanner were included. Exclusion criteria for HC participants were: (i) history or presence of any neurological or psychiatric disorder and (ii) history of substance abuse (including alcohol). Finally, 40 HC participants with MRI at 7T were selected from the atlasing of the basal ganglia (ATAG) project based on image quality and age range (mean age 30.4 ± 12.1 years, range 19–59 years; 21 male). Detailed description of this dataset can be found in Forstmann, Keuken.^23^ None of the ATAG participants suffered from neurological, psychiatric, or somatic diseases.

### MRI acquisition

For each participant, the 3T‐MRI scan data were acquired on a Siemens Prisma^FIT^ scanner using an anatomical magnetization‐prepared rapid acquisition with gradient‐echo (MPRAGE) sequence, isotropic resolution 1 × 1 ×1 mm^3^, TE/TR = 2.21/2250 ms, flip angle = 9°, TI = 900, matrix size = 256 × 256.

7T‐MRI data were acquired with a Siemens whole‐body MAGNETOM 7T scanner (Siemens Healthcare, Erlangen, Germany) using a magnetization‐prepared two rapid acquisition with gradient‐echo sequence with bias field correction (MP2RAGE; isotropic resolution 0.7 × 0.7 × 0.7 mm, TE/TR—2.47/5000 ms, flip angle #1/#2 = 5°/3°, TI #1/#2 = 900/2750, matrix size = 320 × 320 × 240, scan duration 10 min). Data acquisition and online image reconstruction were performed with a vendor‐supplied Works‐in‐progress (WIP) MP2RAGE package. To minimize the effects of B1 inhomogeneity, dielectric pads^24^ were placed between the subject’s head and the coil, positioned in correspondence with temporal and occipital lobes, that is, the brain areas most affected by such inhomogeneity in a volume transmit coil. Further imaging details can be found in De Santis, Bastiani.^7^

7T‐MRI data from the ATAG cohort were similarly acquired using a standardized MP2RAGE imaging protocol on the same 7T Siemens MAGNETOM scanner with the following parameters: voxel size = 0.7 × 0.7 × 0.7 mm, TE/TR = 2.45/5 ms, flip angle #1/#2 = 5°/3°, TI #1/#2 = 900/2750 ms, 240 slices, and GRAPPA acceleration factor 2, scan duration 10 min 57 s.

MP2RAGE sequences combine two gradient‐recalled echo images acquired at different inversion times to obtain a quantitative T1 map, with spatially uniform tissue contrast.^25^ However, this sequence amplifies the noise outside of the brain and adjacent to the cortical gray matter. To mask this amplified noise, we multiplied the T1 map by a proton density (PD)‐weighted image acquired during the first half of the inversion recovery, which has low voxel intensities in the image background regions.^26^ The product image was then skull‐stripped to generate a brain mask including the brain but excluding the amplified noise regions. The resulting mask was then applied to the original T1 map to segment the brain from the surrounding amplified noise. This noise masked image was used in all subsequent analyses.

### Cortical surface reconstruction and measures

MRI data from each participant in both groups/scanners were automatically processed with FreeSurfer (v6.0; http://surfer.nmr.mgh.harvard.edu). Briefly, structural images were bias field corrected, intensity normalized, and skull stripped with a watershed algorithm, followed by creation of a WM volume for each hemisphere, definition of white (WM/GM boundary) and pial (GM/ cerebrospinal fluid [CSF] boundary) surfaces, and topology correction.^27^ The GM/WM boundary served as reference for the tessellated cortical surfaces. Reconstructed surface boundaries were inspected, and manual editing was performed according to standard FreeSurfer quality control procedures.

For the main analyses, we extracted average morphometric measures for each of the 68 cortical regions in the Desikan‐Killiany atlas.^28^ In order to quantify the differences between 3T‐ and 7T‐MRI, we computed different image quality measures across the whole brain:

- The global CNR was estimated as the ratio of the difference in the mean signal intensity of WM and GM tissue voxels, divided by the standard deviation of the signals:
  $$\text{CNR}=\frac{\text{sqr}\left(\text{mG}-\text{mW}\right)}{\text{Wvar}+\text{Gvar}}$$
  where mG and mW are the mean intensity signal of GM and WM tissues, respectively; Gvar and Wvar are the standard deviations of the two tissues. Higher CNR indicates higher data quality and thus better performance for image pre‐processing involving tissue segmentation. Given that for network analysis, regional cortical surface values are used (see below), a surface‐based CNR (sCNR) was further computed using the same method, where the tissue intensities were mapped to the respective pial and white surfaces.

- The GWc was calculated using FreeSurfer’s “*pctsurfcon*” tool. For this, intensity maps for WM and GM were created using the values of the T1‐weighted signal. Then, WM/GM intensity contrast maps were calculated using the percentage contrast between WM and GM intensities:

$$\text{GWc}=\frac{100*\left(W-G\right)}{0.5*\left(W+G\right)}$$

where W is the WM intensity and G is the GM intensity. Here, W is sampled 1 mm below the white surface, whereas G is sampled 30% into the thickness of the cortex. Therefore, by definition, an increase in GWc is commensurate with an increase in contrast between the GM and the WM tissues.^29^

CT was calculated as the closest distance from the white to the pial surface at each vertex. These maps were created using spatial intensity gradients across tissue classes and, therefore, not simply reliant on absolute signal intensity.

### Network analysis

The CT and GWc morphometric networks of each group were computed as the pairwise covariance similarity (Pearson's correlation) across cortical regions. This yielded a separate covariance matrix (N × N, where N represents the 68 cortical areas) for MS’s and HC’s 3T‐ and 7T‐MRI data. The covariance matrices were binarized by applying a minimum density threshold (0.5), determined to guarantee that the reconstructed GM morphometric networks were fully connected and comparable across groups.

To characterize the topological properties of the morphometric networks, several key metrics were calculated at 20 network densities (in 1% density steps), including modularity (Q; measuring the divisibility of a network into modules and representing the relation between intramodular and intermodular connections), the average clustering coefficient (Cp) (pertaining to the number of existing connections between a node i and its neighbors divided by all possible connections), and the characteristic path length (Lp; the average distance of the shortest path involving all node pairs within the network, in which the shortest path represents the number of edges that connect two nodes).^30^ A brain network that features efficient transfer of parallel information at a relatively low cost (higher Cp yet similar Lp compared to the null random networks) is known as a small‐world network.^31^, ^32^ To measure the network’s small‐worldness (σ), the Cp and Lp are normalized by the corresponding average Cp and Lp of matching random networks (*N* = 100) leading to the metrics: normalized Cp (γ = Cp/Crandp) and normalized Lp (λ = Lp/Lrandp). A small‐world network fulfils the following criteria: γ > 1, λ ~ 1, and σ = γ/λ > 1. Null random networks were generated from covariance matrices that match the distributional features of the observed covariance matrix.^33^

### Statistical analysis

Group differences in SNR, CNR, sCNR, CT, and GWc were assessed using analysis of covariance (ANCOVA) under the general linear model (GLM). CT and GWc GLM‐ANCOVAs were performed across the 68 cortical parcels (34 per hemisphere) as dependent variables and group as between‐subject factor. All models included age, gender, and MRI‐scanner as covariates. GLM‐ANCOVA post hoc analyses were corrected using the Tukey–Kramer test (significant level = 0.05). Correction for multiple comparisons across the 68 bilateral cortical regions was performed using the false discovery rate (FDR) at 95% confidence. We further examined how the morphometric measures CT and GWc regionally co‐vary across the cortical mantle between 3T and 7T using a linear regression model.

Group differences in network topology across metrics (as dependent variables) and group (as between‐subject factor) were tested with a GLM‐ANOVA. Post hoc analyses were corrected using the Tukey–Kramer test (significant level = 0.05). Correction for multiple comparisons across network metrics and groups was performed using the false discovery rate (FDR) at 95% confidence.

All statistical analyses were conducted in MATLAB (R2017b).

## Results

No differences in age (*P* = 0.081, *t* = 1.6) or gender (*P* = 0.65, chi‐squared = 1.7) distributions were attested between groups. Comparing to HC, at 3T, MS patients showed increased white matter lesion (WML) volume (*P* = 0.03, *t* = 1.9) and only a trend for reduced brain parenchymal volume (BPV; *P* = 0.053, *t* = 1.6), whereas at 7T, MS patients showed both increased WML volume (*P* = 0.02, *t* = 2.1) and reduced BPV (*P* = 0.02, *t* = 2.2). Within MS patients, no differences in WML volume (*P* = 0.3, *t* = 0.4) but reduced BPV (*P* = 0.007, *t* = 2.9) appeared when comparing 7T‐ with 3T‐MRI. Within HC, no differences in WML nor BPV were attested between 3T‐ and 7T‐MRI.

### Comparison of SNR, CNR, and sCNR between 7T‐ and 3T‐MRI

**Figure 1:**
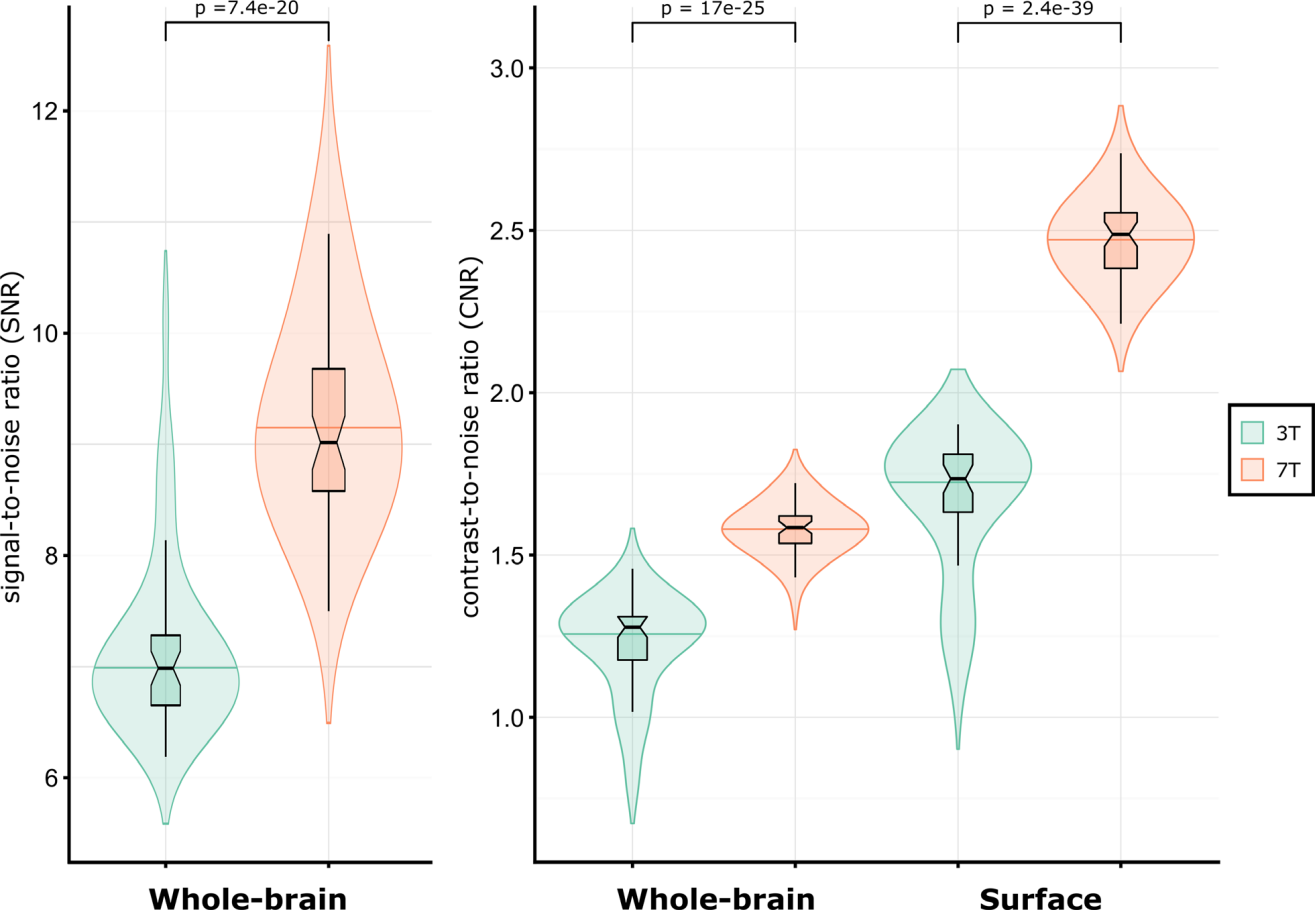
Differences in signal‐to‐noise ratio (SNR), contrast‐to‐noise ratio (CNR), and surface‐based CNR (sCNR) between 3T‐ and 7T‐MRI data.

Compared to 3T‐data, 7T‐MRI had increased global SNR (*P* = 7.4e‐20, *t* = 11.7). Consistently, 7T‐data had clearer tissue boundaries, evidenced by increased global CNR (*P* = 1.1e‐25, *t* = 14.6) and sCNR (*P* = 2.4e‐39, *t* = 22.6) with respect to 3T (Fig. 1).

### Morphometric comparison between 7T‐ and 3T‐MRI

**Figure 2:**
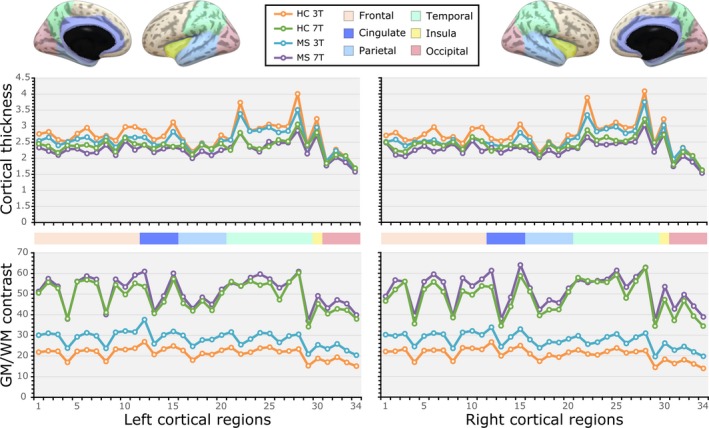
Comparison of the average gray matter (GM)‐to‐white matter (WM) percent contrast (GWc) and cortical thickness (CT) across 34 regions of the left and right hemispheres between 3T‐ and 7T‐MRI data of healthy control (HC) participants and multiple sclerosis (MS) patients. The corresponding statistics for each MRI morphometric measure across cortical regions of each hemisphere are shown in supplementary Tables 1 and 2. The region numbers in the X‐axis have an exact correspondence to the regional order in supplementary Tables 1 and 2. For reference, the hemispheric regions are ordered and represented according to the lobule they form part of (frontal = beige colour; cingulate = dark blue; parietal = light blue; temporal = light green; insula = yellow; and occipital = pink).

Regionally, the GLM‐ANCOVA evidenced increased GWc in 7T‐MRI across the cortex in the two hemispheres (Fig. 2 and Tables S1 and S2). Differences in GWc across groups reached statistical significance in several cortical areas as HC 3T < MS 3T < HC 7T < MS 7T. The GLM‐ANCOVA for CT showed that at 7T significantly reduced values are estimated compared to 3T (HC 3T> MS 3T> HC 7T> MS 7T). Both morphometric measures of cortical integrity yielded similar distribution patterns across cortical regions independently of the magnetic field, 3T or 7T in both groups (Fig. 2), highlighting the robustness of the acquisitions and processing pipeline against potential field strength‐related effects.

**Figure 3:**
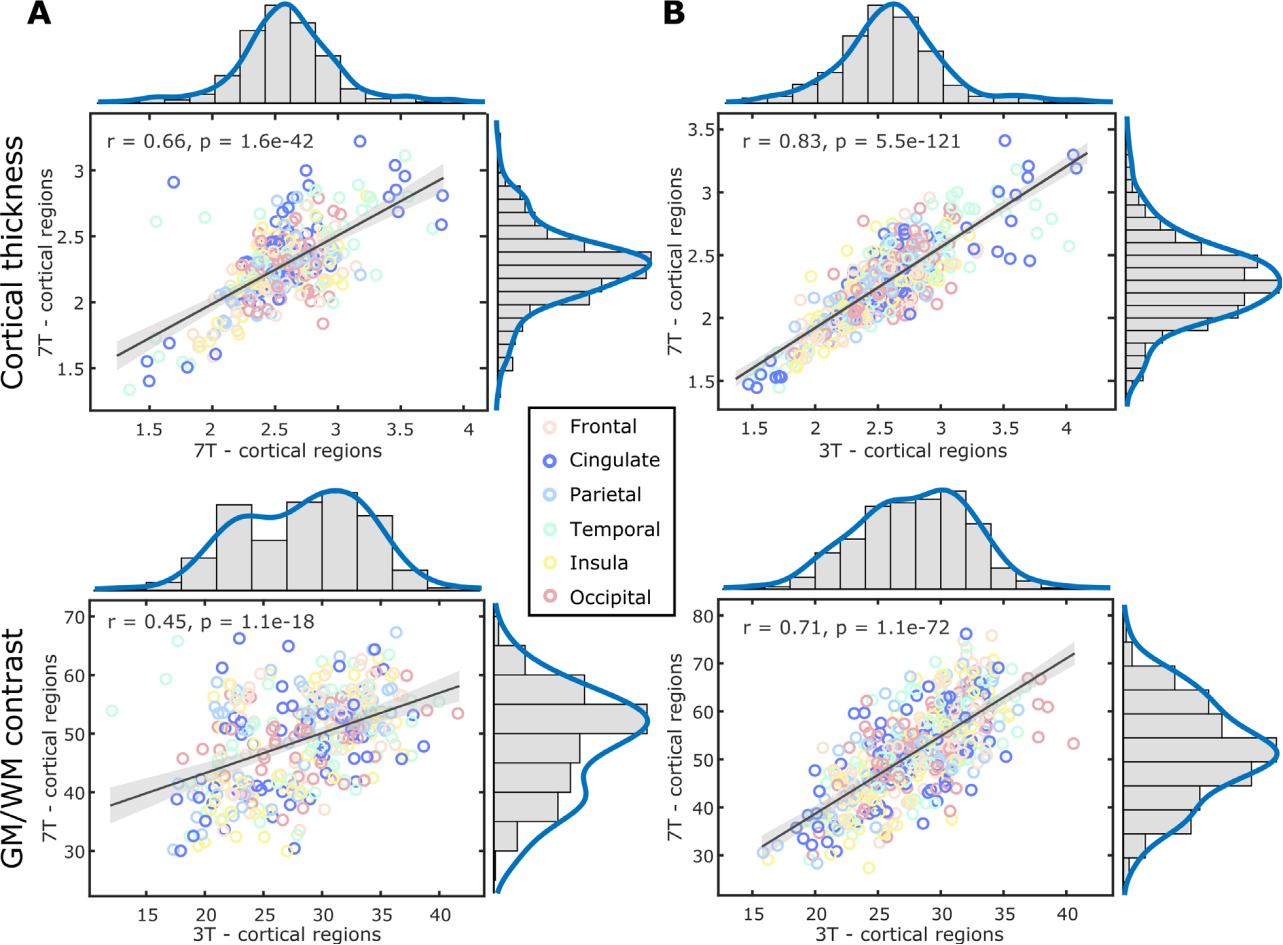
Regional correspondence of grey matter (GM)‐to‐white matter (WM) percent contrast (GWc) and cortical thickness (CT) with distribution plots for the 68 cortical regions between 3T‐ and 7T‐MRI data of healthy controls (A) and multiple sclerosis patients (B). For reference, the hemispheric regions are ordered and represented according to the lobule they form part of (frontal = beige colour; cingulate = dark blue; parietal = light blue; temporal = light green; insula = yellow; and occipital = pink).

Furthermore, the regression analyses evidenced high regional correspondence for both CT (*P* = 1.6e‐42, *r* = 0.66) and GWc (*P* = 1.1e‐18, *r* = 0.45) in HC, and CT (*P* = 5.5e‐121, *r* = 0.83) and GWc (*P* = 1.1e‐72, *r* = 0.71) in MS, indicating that regions with the highest and lowest values across the cortex were the same regions at 3T and 7T (Fig. 3).

### Comparison of GM morphometric network metrics between 7T‐ and 3T‐MRI

**Figure 4:**
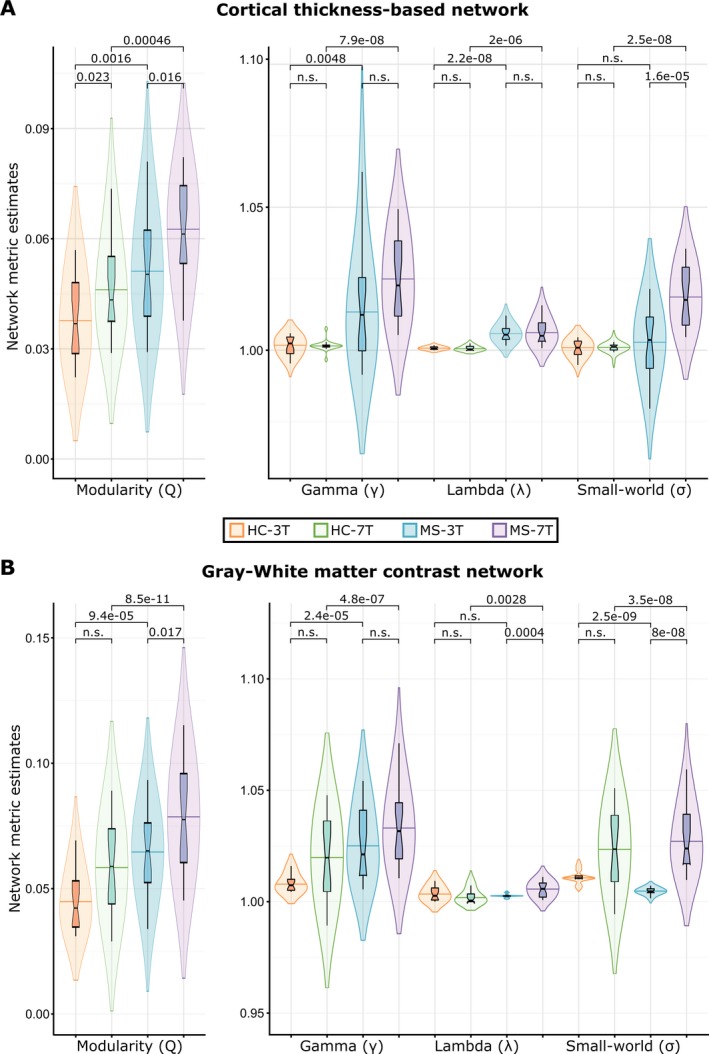
Group differences in network measures: modularity (Q), normalized clustering coefficient (γ), normalized path length (λ) and small‐world (σ) of morphometric networks derived from 3 Tesla (3T)‐ and 7 Tesla (7T)‐MRI datasets of healthy controls (HC) multiple sclerosis (MS) patients for networks based on cortical thickness (A) and grey matter‐to‐white matter percent contrast (B). p‐values are corrected for multiple comparisons across groups and network parameters using FDR at 95% confidence level. In all plots groups are ordered from left to right are: HC at 3T (orange), HC at 7T (green), MS at 3T (blue), and MS at 7T (purple).

For the CT‐based network, overall effects of MS and MRI magnetic field were shown as increased modularity (F(3,88) = 12.6, pFDR = 3.2e‐07), normalized clustering coefficient (F(3,88) = 17.5, pFDR = 2.8e‐09), normalized path length (F(3,91) = 30.7, pFDR = 6.7e‐14), and small‐world (F(3,88) = 25.3, pFDR = 4e‐12) (mostly HCs 3T < controls 7T < MS 3T < MS 7T). The same effect appeared for the GWc‐based network with increased modularity (F(3,88) = 9.2, pFDR = 1.1e‐05), normalized clustering coefficient (F(3,88) = 5.1, pFDR = 0.0013), normalized path length (F(3,88) = 5.9, pFDR = 5.4e‐05), and small‐world (F(3,88) = 6.3, pFDR = 3.3e‐04) (tendency: controls 3T ≤ controls 7T < MS 3T < MS 7T). Hence, both morphometric networks likely depict a similar underlying process (Fig. 4).

## Discussion

Here, we evaluate whether MRI at ultra‐high magnetic fields (i.e., 7T) has an impact on the characterization of cortical morphometric network topology, as derived from cortical thickness and gray‐to‐white matter contrast attributes. Our results evidence high regional correspondence of CT and GWc in controls and MS patients between 3T‐ and 7T‐data across the cortical mantle. Both MRI data attested robust group differences between MS patients and controls at 3T and consistent, though less pronounced, group differences at 7T. Regarding network properties, increased modularity and normalized clustering coefficient in MS patients compared to HC, accompanied by increased normalized path lengths and small‐worldness, are detectable irrespective of the magnetic field for CT but only at 7T for GWc. Altogether, these findings show the current utility of 3T, despite the proposed advantages of 7T, to reliably depict widespread brain reorganization processes in MS patients at the level of brain networks, while discrete structural morphometrics may be better detected by 7T‐MRI.

GM pathology has emerged as one of the pivotal contributors of long‐term clinical disability in MS^3^, ^34^ and much effort has been put into describing the structural aspects of GM pathology that might provide information about underlying neuro‐axonal damage. Thus, there is urgency to use stronger UHF‐MRI techniques and establish non‐invasive neuroimaging biomarkers of GM pathology. We have recently shown the advantages of 7T‐MRI in detecting microstructural tissue integrity of normal appearing GM and WM^7^ and meningeal inflammation.^10^ While most of the previous studies using 7T‐MRI focused on detection of cortical lesions^7^, ^9^, ^10^, ^13^, ^35^ here, we analyzed proxies of cortical GM tissue degeneration, namely CT and GWc. Moving from 3T‐ to 7T‐MRI evidenced clear and systematic patterns of cortical thinning, which are topographically related to increased GWc in MS patients. In healthy individuals, a similar topography when comparing CT between 7T‐ and 3T‐MRI was further evidenced in our study. These patterns appear to be robust to the selection of MRI sequence.^11^, ^26^ Suggesting improved tissue delineation between GM and WM during MRI processing, and extension of recognizable disease‐related abnormalities that could be detected across the cortical mantle of MS patients. Thus, the results are likely to be generalizable to further populations.

Regional variations in CT reflect the degree of neuronal loss across cortical layers, whereas GWc relates to myelin content within the GM/WM boundaries,^19^ both being regarded as sensitive measures for mapping the spatial vulnerability of GM tissue. In our study, the most notorious regional group and magnetic field differences appeared in the frontal, cingulate, and temporal cortices. These specific brain regions have been proved to be highly relevant for the clinical and pathological manifestation of MS.^36^, ^37^, ^38^ Early studies have proposed that the sensitivity of GWc to GM damage can overcome that of CT.^19^, ^29^ The current results, however, shed light on the possible closer association and impact of GWc measurements over CT, which may be overestimated in regions with reduced GWc, as suggested by the inverse pattern between CT and GWc, particularly at some regions of the occipital lobe that not differ between MS and HC at 3T but at 7T. According with our results, CT and GWc have been suggested to be not entirely independent measures.^29^ Despite operating with a moderate sample size, we were able to obtain consistent results on the variations in field strengths in sensing the alterations of CT and GWc, and hence also GM pathology, and network reorganization. However, the value of both morphometric measures derived from 7T‐MRI should be further validated in larger MS cohorts. As brain MRI has become an indispensable tool in the diagnosis and monitoring of MS pathology, broad implementation of 7T‐MRI platforms may translate into increased reliability in the assessment of incipient GM pathology with indisputable benefits for research and clinical practice. Moreover, the recently introduced graph theory network perspective opens the possibility to depict cortical reorganization in MS patients beyond established measures of brain or cortical integrity even in patients with not yet measurable GM atrophy over time.^20^

In vivo portraying MS‐induced GM pathology is limited by the threshold of detectability inherent to the technical constraints of MRI. Consequently, a growing body of evidence offered by network neuroscience highlights the relevance of network‐based approaches in understanding the fundamental principles and pathophysiological mechanisms of network responses elicited by GM neurodegeneration. Modeling brain networks based on 3T‐MRI data has become a widely accepted tool to describe tissue reorganization occurring beyond the MS pathology recognizable by common MRI morphometric measures.^20^, ^39^, ^40^ Here, morphometric networks reconstructed on CT and GWc from 7T‐ and 3T‐MRI data were characterized by the same directionality of network metrics: higher modularity and normalized clustering coefficient in MS patients, and preserved small‐world characteristics. Yet, higher values in network metrics from 7T‐data may be directly related to the differences in CT and GWc obtained between 7T‐ and 3T‐scanners. As network modules exhibit high intramodular dependencies and high intermodular independencies, increased modularity suggests increased segregation of neuronal circuitries within the cortical GM morphometric networks in MS.^20^, ^41^ Reorganization of cortical networks following disease onset indicates to act as a compensatory response of cortical GM tissue, associated with long‐term clinical outcomes in MS.^20^, ^41^, ^42^ Besides current measures of cortical GM integrity, network measures, closely reflecting ongoing tissue damage and repair processes, are promising markers for sensibly depicting disease progression.

Altogether, brain morphometric attributes inferred from cortical GM pathology from UHF‐MRI might provide early red flags of incipient neurodegeneration, and be further incorporated into longitudinal frameworks to track disease progression. By probing advantage of 7T‐MRI to characterize cortical GM integrity through CT and GWc, we further evidenced that these measures serve to meaningfully build morphometric networks. Thus, 7T‐MRI outperforms 3T‐MRI by yielding higher spatial resolutions (higher SNR, CNR, and GWc) that emerge as more precise appreciation of abnormal cortical GM morphometry (lower CT), as well sensitively portraying cortical GM network topology organization.

Some limitations should be noted. First, despite the cohort of HC has been selected to precisely match the acquisition parameters of the MRI (3T) data in MS, while reducing the margin of error for the measured MRI parameters by increasing the sample size in the HC, this increase in sample size was not possible in MS patients given the costly dual 3T‐ and 7T‐MRI acquisitions. This also resulted in distinct (although not statistically different) age means between MS and HC. To prevent the reported differences to be possibly driven by remaining aging or gender effects, age and gender were included into all analyses as possible confounders. Another limitation of the study related to the low number of included patients is the impossibility to infer the effects of disease‐modifying drugs on cortical GM properties. This could be achieved by conducting longitudinal studies with larger age‐matched samples, including both 3T‐ and 7T‐data. Last, in our study, we included MS patients with short disease duration. Furthermore, longitudinal studies in larger cohorts evaluating both later stage relapsing‐remitting and progressive MS forms will provide further clarification on how disease severity, progression, and medication influence different MRI‐derived morphometric and network measures.

Neuroimaging has evolved into a key modality enabling *in vivo* exploration of disease‐driven processes and depiction of brain tissue dynamics at the macro‐ and microstructural levels. The advent of UHF‐MRI platforms, including better spatial resolution and enhanced SNR and CNR, proved better tissue differentiation, significantly advancing the detectability of compartmentalized tissue responses and, hence, provide an accurate characterization of the continuum of MS pathology in spatial dimensions. However, in order to take clinical advantage offered by UHF‐MRI technology, progress should be made on current technical constraints, such as image non‐uniformities, reduction of specific absorption rate, and susceptibility‐related artifacts.^43^ In all, together with the high cost of UHF MRI scanners, turn UHF imaging not currently clinically feasible. Nevertheless, the employment of UHF‐MRI platforms open a broad range of possibilities for next‐generation clinical and research studies in MS to more sensitively detect morphometric reorganization dynamics within the cortical GM and evaluate discrete correlates of neuroinflammatory and neurodegenerative tissue responses.

## Conclusions

Our data evidence that 7T‐MRI‐based evaluation of cortical microanatomy confers an increased degree of precision to detect and quantify GM pathology, compared to 3T‐MRI. Relying on improved spatial resolution and tissue contrast differences, 7T‐MRI provides more depth for the in vivo characterization of microstructural properties of the cortical mantle as well as of morphometric network topology to be used as proxies of disease‐related cortical GM damage. Large‐scale implementation of 7T‐MRI within studies of the human connectome offers the opportunity to carry out a detailed assessment of disease progression, prognostic patient stratification, and treatment effects focusing on tissue damage and repair.

## Conflict of interests

None of the authors have any conflict of interest to disclose.

## Supporting information


**Table S1**. Comparison of the average cortical thickness (CT) across 68 cortical regions between 3T‐ and 7T‐MRI data of MS patients and control individuals (HC).
**Table S2**. Comparison of the average gray‐to‐white percent contrast (GWc) across 68 cortical regions between 3T‐ and 7T‐MRI data of MS patients and control individuals (HC).

## Data Availability

The data that support the findings of this study are available from the corresponding author upon reasonable request. ATAG data are freely available online (https://www.nitrc.org/projects/atag/ and https://datadryad.org/stash/dataset/doi:10.5061/dryad.fb41s).

## References

[1] Calabrese M , Magliozzi R , Ciccarelli O , et al. Exploring the origins of grey matter damage in multiple sclerosis. Nat Rev Neurosci 2015;16:147–158. 25697158 10.1038/nrn3900

[2] Kramer J , Bruck W , Zipp F , et al. Imaging in mice and men: Pathophysiological insights into multiple sclerosis from conventional and advanced MRI techniques. Prog Neurogibol 2019;30:101663. 10.1016/j.pneurobio.2019.101663 31374243

[3] Jacobsen C , Hagemeier J , Myhr K‐M , et al. Brain atrophy and disability progression in multiple sclerosis patients: a 10‐year follow‐up study. J Neurol Neurosurg Psychiatry 2014;85:1109–1115. 24554101 10.1136/jnnp-2013-306906

[4] Obusez EC , Lowe M , Oh S‐H , et al. 7T MR of intracranial pathology: Preliminary observations and comparisons to 3T and 1.5 T. NeuroImage 2018;168:459–476. 27915116 10.1016/j.neuroimage.2016.11.030

[5] Sati P . Diagnosis of multiple sclerosis through the lens of ultra‐high‐field MRI. J Magn Reson 2018;291:101–109. 29705032 10.1016/j.jmr.2018.01.022 PMC6022748

[6] Fartaria MJ , Sati P , Todea A , et al. Automated detection and segmentation of multiple sclerosis lesions using ultra‐high‐field MP2RAGE. Invest Radiol 2019;54:356–364. 30829941 10.1097/RLI.0000000000000551 PMC6499666

[7] De Santis S , Bastiani M , Droby A , et al. Characterizing microstructural tissue properties in multiple sclerosis with diffusion MRI at 7 T and 3 T: the impact of the experimental design. Neuroscience 2019;403:17–26. 29631021 10.1016/j.neuroscience.2018.03.048

[8] Absinta M , Sati P , Gaitán MI , et al. Seven‐tesla phase imaging of acute multiple sclerosis lesions: a new window into the inflammatory process. Ann Neurol 2013;74:669–678. 23813441 10.1002/ana.23959 PMC3812397

[9] de Graaf WL , Kilsdonk ID , Lopez‐Soriano A , et al. Clinical application of multi‐contrast 7‐T MR imaging in multiple sclerosis: increased lesion detection compared to 3 T confined to grey matter. Eur Radiol 2013;23:528–540. 22898935 10.1007/s00330-012-2619-7

[10] Kolber P , Droby A , Roebroeck A , et al. A "kissing lesion": In‐vivo 7T evidence of meningeal inflammation in early multiple sclerosis. Mult Scler J 2017;23:1167–1169. 10.1177/1352458516683267 28417657

[11] Lusebrink F , Wollrab A , Speck O . Cortical thickness determination of the human brain using high resolution 3 T and 7 T MRI data. NeuroImage 2013;15:122–131. 10.1016/j.neuroimage.2012.12.016 23261638

[12] Pohmann R , Speck O , Scheffler K . Signal‐to‐noise ratio and MR tissue parameters in human brain imaging at 3, 7, and 9.4 tesla using current receive coil arrays. Magn Reson Med 2016;75:801–809. 25820458 10.1002/mrm.25677

[13] Yao B , Hametner S , van Gelderen P , et al. 7 Tesla magnetic resonance imaging to detect cortical pathology in multiple sclerosis. PLoS ONE 2014;9:e108863. 25303286 10.1371/journal.pone.0108863 PMC4193749

[14] Duyn JH . The future of ultra‐high field MRI and fMRI for study of the human brain. NeuroImage 2012;62:1241–1248. 22063093 10.1016/j.neuroimage.2011.10.065 PMC3389184

[15] Amiri H , de Sitter A , Bendfeldt K , et al. Urgent challenges in quantification and interpretation of brain grey matter atrophy in individual MS patients using MRI. Neuroimage Clin 2018;19:466–475. 29984155 10.1016/j.nicl.2018.04.023 PMC6030805

[16] Storelli L , Rocca MA , Pagani E , et al. Measurement of Whole‐Brain and gray matter atrophy in multiple sclerosis: assessment with MR imaging. Radiology 2018;288:554–564. 29714673 10.1148/radiol.2018172468

[17] Rocca MA , Comi G , Filippi M . The role of T1‐weighted derived measures of neurodegeneration for assessing disability progression in multiple sclerosis. Front Neurol 2017;8:433. 28928705 10.3389/fneur.2017.00433 PMC5591328

[18] Righart R , Schmidt P , Dahnke R , et al. Volume versus surface‐based cortical thickness measurements: a comparative study with healthy controls and multiple sclerosis patients. PLoS ONE 2017;12:e0179590. 28683072 10.1371/journal.pone.0179590 PMC5500013

[19] Vidal‐Pineiro D , Walhovd KB , Storsve AB , et al. Accelerated longitudinal gray/white matter contrast decline in aging in lightly myelinated cortical regions. Hum Brain Mapp 2016;37:3669–3684. 27228371 10.1002/hbm.23267 PMC6867532

[20] Fleischer V , Koirala N , Droby A , et al. Longitudinal cortical network reorganization in early relapsing‐remitting multiple sclerosis. Ther Adv Neurol Disord 2019;12:1756286419838673. 31040880 10.1177/1756286419838673 PMC6482642

[21] Kocevar G , Stamile C , Hannoun S , et al. Graph theory‐based brain connectivity for automatic classification of multiple sclerosis clinical courses. Front Neurosci 2016;10:478. 27826224 10.3389/fnins.2016.00478 PMC5078266

[22] Fleischer V , Radetz A , Ciolac D , et al. Graph theoretical framework of brain networks in multiple sclerosis: a review of concepts. Neuroscience 2019;1:35–53. 10.1016/j.neuroscience.2017.10.033 29101079

[23] Forstmann BU , Keuken MC , Schafer A , et al. Multi‐modal ultra‐high resolution structural 7‐Tesla MRI data repository. Scientific Data 2014;1:140050. 25977801 10.1038/sdata.2014.50 PMC4421933

[24] Teeuwisse W , Brink W , Haines K , Webb A . Simulations of high permittivity materials for 7 T neuroimaging and evaluation of a new barium titanate‐based dielectric. Magn Reson Med 2012;67:912–918. 22287360 10.1002/mrm.24176

[25] Marques JP , Kober T , Krueger G , et al. MP2RAGE, a self bias‐field corrected sequence for improved segmentation and T1‐mapping at high field. NeuroImage 2010;49:1271–1281. 19819338 10.1016/j.neuroimage.2009.10.002

[26] Fujimoto K , Polimeni JR , van der Kouwe AJW , et al. Quantitative comparison of cortical surface reconstructions from MP2RAGE and multi‐echo MPRAGE data at 3 and 7 T. NeuroImage 2014;15:60–73. 10.1016/j.neuroimage.2013.12.012 PMC4035370 24345388

[27] Fischl B . FreeSurfer. NeuroImage 2012;62:774–781. 22248573 10.1016/j.neuroimage.2012.01.021 PMC3685476

[28] Desikan RS , Ségonne F , Fischl B , et al. An automated labeling system for subdividing the human cerebral cortex on MRI scans into gyral based regions of interest. NeuroImage 2006;31:968–980. 16530430 10.1016/j.neuroimage.2006.01.021

[29] Salat DH , Lee SY , van der Kouwe AJ , et al. Age‐associated alterations in cortical gray and white matter signal intensity and gray to white matter contrast. NeuroImage 2009;48:21–28. 19580876 10.1016/j.neuroimage.2009.06.074 PMC2750073

[30] Rubinov M , Sporns O . Complex network measures of brain connectivity: uses and interpretations. NeuroImage 2010;52:1059–1069. 19819337 10.1016/j.neuroimage.2009.10.003

[31] Achard S , Bullmore E . Efficiency and cost of economical brain functional networks. PLoS Comput Biol 2007;3:e17. 17274684 10.1371/journal.pcbi.0030017 PMC1794324

[32] Watts DJ , Strogatz SH . Collective dynamics of 'small‐world' networks. Nature 1998;393:440–442. 9623998 10.1038/30918

[33] Zalesky A , Fornito A , Bullmore E . On the use of correlation as a measure of network connectivity. NeuroImage 2012;60:2096–2106. 22343126 10.1016/j.neuroimage.2012.02.001

[34] Harrison DM , Roy S , Oh J , et al. Association of cortical lesion burden on 7‐T magnetic resonance imaging with cognition and disability in multiple sclerosis. JAMA Neurol 2015;72:1004–1012. 26192316 10.1001/jamaneurol.2015.1241 PMC4620027

[35] Treaba CA , Granberg TE , Sormani MP , et al. Longitudinal characterization of cortical lesion development and evolution in multiple sclerosis with 7.0‐T MRI. Radiology 2019;291:740–749. 30964421 10.1148/radiol.2019181719 PMC6543899

[36] Geisseler O , Pflugshaupt T , Bezzola L , et al. The relevance of cortical lesions in patients with multiple sclerosis. BMC Neurol 2016;16:204. 27769199 10.1186/s12883-016-0718-9 PMC5073896

[37] Steenwijk MD , Geurts JJ , Daams M , et al. Cortical atrophy patterns in multiple sclerosis are non‐random and clinically relevant. Brain 2016;139(Pt 1):115–126. 26637488 10.1093/brain/awv337

[38] Trapp BD , Vignos M , Dudman J , et al. Cortical neuronal densities and cerebral white matter demyelination in multiple sclerosis: a retrospective study. Lancet Neurol 2018;17:870–884. 30143361 10.1016/S1474-4422(18)30245-X PMC6197820

[39] Charalambous T , Tur C , Prados F , et al. editors. The relationship between network measures and magnetic resonance imaging metrics in multiple sclerosis. Mult Scler J 2017:SAGE.

[40] Tur C , Eshaghi A , Altmann DR , et al. Structural cortical network reorganization associated with early conversion to multiple sclerosis. Sci Rep 2018;8:10715. 30013173 10.1038/s41598-018-29017-1 PMC6048099

[41] Ciolac D , Luessi F , Gonzalez‐Escamilla G , et al. Selective brain network and cellular responses upon dimethyl fumarate immunomodulation in multiple sclerosis. Front Immunol 2019;10:1779. 31417557 10.3389/fimmu.2019.01779 PMC6682686

[42] Tur C , Kanber B , Eshaghi A , et al. Clinical relevance of cortical network dynamics in early primary progressive MS. Multiple Sclerosis Journal 2019;1352458519831400. 10.1177/1352458519831400 30799709

[43] Karamat MI , Darvish‐Molla S , Santos‐Diaz A . Opportunities and challenges of 7 tesla magnetic resonance imaging: a review. Crit Rev Biomed Eng 2016;44(1–2):73–89. 27652452 10.1615/CritRevBiomedEng.2016016365

