# Supplementary material for: Gray matter network reorganization in multiple sclerosis from 7‐Tesla and 3‐Tesla MRI data
Source: Ann Clin Transl Neurol. 2020 Apr 7;7(4):543–53. doi: 10.1002/acn3.51029 (PMC7187719; doi:10.1002/acn3.51029)
Supplement: Supplementary file 1 — Table S1. Comparison of the average cortical thickness (CT) across 68 cortical regions between 3T‐ and 7T‐MRI data of MS patients and control individuals (HC). Table S2. Comparison of the average gray‐to‐white percent contrast (GWc) across 68 cortical regions between 3T‐ and 7T‐MRI data of MS patients and control individuals (HC). [file ACN3-7-543-s001.docx]

**Supplementary Table 1.** Comparison of the average cortical thickness (CT) across 68 cortical regions between 3T- and 7T-MRI data of MS patients and control individuals (HC).

| Cortical thickness | HC (3T) | HC (7T) | MS (3T) | MS (7T) | Full model | | HC-3T Vs MS-3T | HC-7T Vs MS-7T | HC-3T Vs HC-7T | MS-3T Vs MS-7T |  | HC (3T) | HC (7T) | MS (3T) | MS (7T) | Full model | | HC-3T Vs MS-3T | HC7T Vs MS-7T | HC-3T Vs HC-7T | MS-3T Vs MS-7T |
| --- | --- | --- | --- | --- | --- | --- | --- | --- | --- | --- | --- | --- | --- | --- | --- | --- | --- | --- | --- | --- | --- |
|  | mean ± SD | mean ± SD | mean ± SD | mean ± SD | F_83_ | pFDR | pFDR | pFDR | pFDR | pFDR |  | mean ± SD | mean ± SD | mean ± SD | mean ± SD | F_83_ | pFDR | pFDR | pFDR | pFDR | pFDR |
|  | **Left hemisphere** | | | | | | | | | |  | **Right hemisphere** | | | | | | | | | |
| Caudalmiddlefrontal | 2.76 ± 0.14 | 2.44 ± 0.13 | 2.54 ± 0.14 | 2.33 ± 0.17 | 37.26 | **1.4E-14** | **0.0093** | **1.30E-01** | **7.40E-09** | **0.025** |  | 2.7 ± 0.13 | 2.48 ± 0.18 | 2.5 ± 0.15 | 2.48 ± 0.22 | 13.59 | **4.8E-07** | **0.016** | 0.49 | 1.30E-07 | 0.99 |
| Lateralorbitofrontal | 2.82 ± 0.16 | 2.36 ± 0.14 | 2.65 ± 0.1 | 2.23 ± 0.05 | 71.93 | **1.4E-21** | **0.0068** | **1.50E-03** | **7.40E-09** | **9.50E-06** |  | 2.79 ± 0.18 | 2.22 ± 0.14 | 2.58 ± 0.13 | 2.1 ± 0.09 | 105.44 | **3.9E-26** | **0.0079** | **4.10E-02** | **7.40E-09** | **7.50E-07** |
| Medialorbitofrontal | 2.57 ± 0.16 | 2.16 ± 0.17 | 2.4 ± 0.09 | 2.1 ± 0.13 | 42.56 | **6.3E-16** | **0.0068** | **2.00E-01** | **7.40E-09** | **0.0076** |  | 2.56 ± 0.2 | 2.18 ± 0.16 | 2.38 ± 0.12 | 2.06 ± 0.2 | 30.58 | **8.4E-13** | **0.013** | **1.30E-01** | **7.40E-09** | **0.0076** |
| Paracentral | 2.52 ± 0.17 | 2.39 ± 0.16 | 2.5 ± 0.15 | 2.28 ± 0.16 | 5.84 | **0.0014** | 0.43 | 0.14 | 0.013 | 0.07 |  | 2.56 ± 0.16 | 2.43 ± 0.15 | 2.5 ± 0.2 | 2.25 ± 0.2 | 7.58 | **2.1E-04** | 0.28 | **0.08** | **0.0058** | **0.023** |
| Parsopercularis | 2.77 ± 0.14 | 2.37 ± 0.12 | 2.6 ± 0.13 | 2.29 ± 0.16 | 60.6 | **1.0E-19** | **0.02** | **2.00E-01** | **7.40E-09** | **6.90E-04** |  | 2.74 ± 0.14 | 2.47 ± 0.14 | 2.52 ± 0.16 | 2.37 ± 0.22 | 25.81 | **2.3E-11** | **0.013** | **0.21** | **7.40E-09** | 0.19 |
| Parsorbitalis | 2.95 ± 0.27 | 2.4 ± 0.17 | 2.66 ± 0.15 | 2.15 ± 0.14 | 50.34 | **1.2E-17** | **0.0068** | **2.10E-02** | **7.40E-09** | **3.10E-04** |  | 2.97 ± 0.24 | 2.44 ± 0.17 | 2.56 ± 0.22 | 2.2 ± 0.18 | 50.29 | **1.2E-17** | **0.0068** | **4.10E-02** | **7.40E-09** | **0.007** |
| Parstriangularis | 2.62 ± 0.15 | 2.31 ± 0.15 | 2.45 ± 0.14 | 2.18 ± 0.13 | 32.8 | **2.0E-13** | **0.024** | **7.00E-02** | **7.40E-09** | **0.0064** |  | 2.6 ± 0.15 | 2.37 ± 0.16 | 2.39 ± 0.14 | 2.29 ± 0.21 | 16.15 | **4.6E-08** | **0.012** | **0.23** | **2.70E-08** | 0.52 |
| Precentral | 2.7 ± 0.16 | 2.54 ± 0.11 | 2.65 ± 0.2 | 2.42 ± 0.16 | 12.25 | **1.7E-06** | 0.325 | 0.11 | **3.10E-05** | **0.0092** |  | 2.66 ± 0.15 | 2.48 ± 0.1 | 2.57 ± 0.19 | 2.45 ± 0.2 | 11.88 | **2.3E-06** | 0.22 | 0.4 | **2.60E-06** | 0.3 |
| Rostralmiddlefrontal | 2.55 ± 0.15 | 2.19 ± 0.12 | 2.34 ± 0.11 | 2.1 ± 0.14 | 49.65 | **1.5E-17** | **0.0061** | **1.20E-01** | **7.40E-09** | **0.0064** |  | 2.45 ± 0.14 | 2.26 ± 0.15 | 2.26 ± 0.14 | 2.15 ± 0.15 | 12.9 | **9.1E-07** | **0.013** | **0.11** | **8.00E-07** | 0.41 |
| Superiorfrontal | 2.98 ± 0.19 | 2.62 ± 0.14 | 2.66 ± 0.15 | 2.54 ± 0.17 | 34.39 | **7.9E-14** | **0.0061** | 0.2 | **7.40E-09** | 0.5 |  | 2.91 ± 0.19 | 2.64 ± 0.16 | 2.61 ± 0.13 | 2.54 ± 0.17 | 20.9 | **9.0E-10** | **0.0022** | **0.15** | **7.50E-09** | 0.81 |
| Frontalpole | 2.98 ± 0.34 | 2.43 ± 0.24 | 2.65 ± 0.28 | 2.26 ± 0.13 | 25.03 | **4.0E-11** | **0.025** | **0.041** | **7.40E-09** | 0.08 |  | 2.95 ± 0.3 | 2.51 ± 0.22 | 2.52 ± 0.18 | 2.21 ± 0.2 | 23.28 | **1.4E-10** | **0.0022** | **3.60E-02** | **8.40E-09** | 0.11 |
| Caudalanterior-cingulate | 2.85 ± 0.22 | 2.4 ± 0.22 | 2.65 ± 0.3 | 2.42 ± 0.33 | 23.26 | **1.4E-10** | 0.11 | 0.47 | **7.40E-09** | 0.3 |  | 2.6 ± 0.21 | 2.21 ± 0.17 | 2.44 ± 0.24 | 2.32 ± 0.29 | 22.29 | **3.0E-10** | 0.11 | **0.23** | **7.40E-09** | 0.76 |
| Isthmuscingulate | 2.57 ± 0.22 | 2.29 ± 0.19 | 2.41 ± 0.14 | 2.18 ± 0.15 | 14.06 | **3.1E-07** | 0.032 | 0.13 | **6.20E-07** | 0.2 |  | 2.53 ± 0.18 | 2.32 ± 0.22 | 2.36 ± 0.25 | 2.16 ± 0.12 | 7.24 | **3.0E-04** | 0.11 | **0.041** | **0.00079** | 0.31 |
| Posteriorcingulate | 2.68 ± 0.11 | 2.42 ± 0.18 | 2.44 ± 0.15 | 2.3 ± 0.17 | 20.06 | **1.7E-09** | **0.0093** | **1.10E-01** | **8.50E-09** | 0.3 |  | 2.63 ± 0.15 | 2.4 ± 0.15 | 2.38 ± 0.31 | 2.3 ± 0.28 | 10.54 | **9.1E-06** | 0.08 | **0.23** | **9.00E-06** | 0.81 |
| Rostralanterior-cingulate | 3.12 ± 0.26 | 2.35 ± 0.24 | 2.83 ± 0.12 | 2.35 ± 0.1 | 70.51 | **2.3E-21** | **0.0022** | **5.00E-01** | **7.40E-09** | **0.0028** |  | 3.05 ± 0.21 | 2.36 ± 0.22 | 2.79 ± 0.15 | 2.34 ± 0.22 | 72.79 | **1.2E-21** | **0.0068** | **4.50E-01** | **7.40E-09** | **0.0013** |
| Inferiorparietal | 2.57 ± 0.14 | 2.39 ± 0.13 | 2.51 ± 0.13 | 2.27 ± 0.15 | 17.46 | **1.4E-08** | 0.22 | 0.11 | **3.70E-07** | **0.0032** |  | 2.64 ± 0.14 | 2.35 ± 0.16 | 2.54 ± 0.14 | 2.24 ± 0.15 | 33.44 | **1.3E-13** | 0.11 | **1.10E-01** | **7.40E-09** | **6.90E-04** |
| Postcentral | 2.2 ± 0.11 | 2.11 ± 0.09 | 2.14 ± 0.09 | 2 ± 0.12 | 7.61 | **0.0002** | 0.12 | 0.08 | **0.0032** | 0.06 |  | 2.16 ± 0.14 | 2.08 ± 0.12 | 2.09 ± 0.13 | 2.02 ± 0.09 | 2.65 | 0.06 | 0.16 | 0.13 | 0.08 | 0.76 |
| Precuneus | 2.48 ± 0.13 | 2.4 ± 0.15 | 2.45 ± 0.1 | 2.22 ± 0.16 | 6.09 | **0.0011** | 0.36 | 0.06 | 0.21 | **0.0047** |  | 2.51 ± 0.13 | 2.42 ± 0.17 | 2.47 ± 0.1 | 2.25 ± 0.17 | 5.32 | **2.6E-03** | 0.29 | **0.06** | **0.15** | **0.014** |
| Superiorparietal | 2.28 ± 0.14 | 2.29 ± 0.12 | 2.26 ± 0.11 | 2.09 ± 0.15 | 3.28 | **0.03** | 0.42 | **0.041** | 0.93 | 0.06 |  | 2.28 ± 0.12 | 2.24 ± 0.12 | 2.27 ± 0.12 | 2.09 ± 0.15 | 3.3 | **2.9E-02** | 0.47 | 0.06 | 0.91 | **0.03** |
| Supramarginal | 2.72 ± 0.15 | 2.44 ± 0.13 | 2.54 ± 0.13 | 2.25 ± 0.19 | 33.79 | **1.1E-13** | **0.017** | **6.30E-02** | **7.40E-09** | **7.20E-04** |  | 2.71 ± 0.13 | 2.36 ± 0.13 | 2.55 ± 0.1 | 2.29 ± 0.13 | 58.69 | **2.2E-19** | **0.0079** | **1.60E-01** | **7.40E-09** | **0.0013** |
| Bankssts | 2.57 ± 0.17 | 2.24 ± 0.15 | 2.57 ± 0.26 | 2.33 ± 0.32 | 20.84 | **9.2E-10** | 0.48 | 0.31 | 8.50E-09 | 0.1 |  | 2.68 ± 0.18 | 2.33 ± 0.16 | 2.65 ± 0.16 | 2.31 ± 0.21 | 29.61 | **1.5E-12** | 0.37 | 0.44 | **7.40E-09** | **0.0035** |
| Entorhinal | 3.73 ± 0.35 | 2.77 ± 0.22 | 3.38 ± 0.33 | 2.78 ± 0.21 | 69.73 | **2.9E-21** | **0.032** | **4.80E-01** | **7.40E-09** | **0.0028** |  | 3.88 ± 0.28 | 2.85 ± 0.35 | 3.34 ± 0.3 | 2.65 ± 0.34 | 63.78 | **2.6E-20** | **0.0068** | **1.50E-01** | **7.40E-09** | **2.40E-03** |
| Fusiform | 2.86 ± 0.14 | 2.37 ± 0.13 | 2.84 ± 0.1 | 2.35 ± 0.14 | 98.67 | **2.4E-25** | 0.39 | **4.50E-01** | **7.40E-09** | **2.50E-07** |  | 2.89 ± 0.13 | 2.53 ± 0.12 | 2.83 ± 0.15 | 2.43 ± 0.13 | 67.81 | **5.9E-21** | 0.22 | **1.00E-01** | **7.40E-09** | **6.00E-07** |
| Inferiortemporal | 2.93 ± 0.14 | 2.28 ± 0.19 | 2.87 ± 0.15 | 2.2 ± 0.11 | 108.22 | **2.3E-26** | 0.28 | **1.50E-01** | **7.40E-09** | **2.50E-07** |  | 2.95 ± 0.14 | 2.63 ± 0.15 | 2.91 ± 0.14 | 2.42 ± 0.1 | 43.61 | **3.7E-16** | 0.33 | **6.70E-03** | **7.40E-09** | **4.60E-07** |
| Middletemporal | 3.05 ± 0.16 | 2.35 ± 0.13 | 2.96 ± 0.25 | 2.52 ± 0.23 | 117.11 | **5.7E-27** | 0.27 | **1.20E-01** | **7.40E-09** | **7.80E-05** |  | 3.11 ± 0.16 | 2.52 ± 0.13 | 2.97 ± 0.19 | 2.45 ± 0.18 | 109.52 | **2.3E-26** | 0.11 | **2.30E-01** | **7.40E-09** | **2.80E-07** |
| Parahippocampal | 3 ± 0.3 | 2.56 ± 0.23 | 2.8 ± 0.13 | 2.47 ± 0.2 | 20.14 | **1.6E-09** | **0.016** | 0.23 | **8.50E-09** | 0.1 |  | 2.95 ± 0.29 | 2.51 ± 0.27 | 2.77 ± 0.14 | 2.48 ± 0.16 | 18.6 | **5.6E-09** | **0.032** | **0.41** | **1.10E-08** | 0.18 |
| Superiortemporal | 2.99 ± 0.18 | 2.51 ± 0.13 | 2.85 ± 0.14 | 2.49 ± 0.15 | 67.26 | **6.8E-21** | 0.053 | **4.00E-01** | **7.40E-09** | **4.50E-04** |  | 2.97 ± 0.17 | 2.65 ± 0.14 | 2.85 ± 0.17 | 2.5 ± 0.17 | 29.69 | **1.5E-12** | 0.11 | **8.00E-02** | **7.40E-09** | **0.0013** |
| Temporalpole | 4.01 ± 0.28 | 3.03 ± 0.29 | 3.51 ± 0.35 | 2.86 ± 0.2 | 73.72 | **1.0E-21** | **0.013** | **1.10E-01** | **7.40E-09** | **0.0018** |  | 4.08 ± 0.29 | 3.18 ± 0.27 | 3.74 ± 0.24 | 3.03 ± 0.38 | 64.35 | **2.2E-20** | **0.013** | **2.30E-01** | **7.40E-09** | **5.00E-04** |
| Transversetemporal | 2.43 ± 0.21 | 2.4 ± 0.22 | 2.38 ± 0.2 | 2.14 ± 0.21 | 2.09 | 0.11 | 0.33 | 0.041 | 0.99 | 0.2 |  | 2.44 ± 0.19 | 2.47 ± 0.22 | 2.37 ± 0.18 | 2.19 ± 0.16 | 2.17 | 0.1 | 0.27 | **0.023** | 0.77 | 0.34 |
| Insula | 3.23 ± 0.14 | 2.78 ± 0.15 | 2.96 ± 0.16 | 2.73 ± 0.15 | 72.68 | **1.2E-21** | **0.0079** | **2.60E-01** | **7.40E-09** | **0.0092** |  | 3.21 ± 0.15 | 2.7 ± 0.16 | 3.02 ± 0.15 | 2.74 ± 0.18 | 78.01 | **2.3E-22** | **0.019** | **3.60E-01** | **7.40E-09** | **0.0039** |
| Cuneus | 1.9 ± 0.17 | 1.84 ± 0.14 | 1.91 ± 0.08 | 1.77 ± 0.12 | 1.97 | 0.13 | 0.42 | 0.15 | 0.5 | 0.3 |  | 1.9 ± 0.17 | 1.79 ± 0.18 | 1.96 ± 0.11 | 1.74 ± 0.12 | 4.39 | **7.7E-03** | 0.17 | 0.23 | 0.1 | **0.1** |
| Lateraloccipital | 2.27 ± 0.14 | 2.1 ± 0.11 | 2.23 ± 0.09 | 2.03 ± 0.07 | 18.13 | **8.2E-09** | 0.26 | **0.09** | **9.20E-08** | **0.0082** |  | 2.33 ± 0.15 | 2.18 ± 0.12 | 2.32 ± 0.12 | 2.06 ± 0.12 | 12.65 | **1.1E-06** | 0.44 | **0.06** | **0.00013** | **0.0022** |
| Lingual | 2.05 ± 0.14 | 2.07 ± 0.12 | 2 ± 0.11 | 1.87 ± 0.15 | 2.84 | **0.048** | 0.24 | **0.041** | 0.77 | 0.2 |  | 2.06 ± 0.13 | 2.06 ± 0.14 | 2.02 ± 0.13 | 1.88 ± 0.14 | 2.09 | 0.11 | 0.29 | **0.041** | 0.99 | 0.28 |
| Pericalcarine | 1.62 ± 0.15 | 1.7 ± 0.13 | 1.64 ± 0.12 | 1.58 ± 0.09 | 2.63 | 0.06 | 0.37 | **0.041** | 0.06 | 0.8 |  | 1.59 ± 0.12 | 1.63 ± 0.17 | 1.62 ± 0.1 | 1.53 ± 0.07 | 0.81 | 0.49 | 0.33 | **0.046** | 0.81 | 0.73 |

Mean regional values are presented ± standard deviation (SD). Bold letters indicate significant (p < 0.05, corrected for multiple comparisons using FDR) differences.

**Supplementary Table 2.** Comparison of the average gray-to-white percent contrast (GWc) across 68 cortical regions between 3T- and 7T-MRI data of MS patients and control individuals (HC).

| GM/WM contrast | HC (3T) | HC (7T) | MS (3T) | MS (7T) | Full model | | HC-3T Vs MS-3T | HC-7T Vs MS-7T | HC-3T Vs HC-7T | MS-3T Vs MS-7T |  | HC (3T) | HC (7T) | MS (3T) | MS (7T) | Full model | | HC-3T Vs MS-3T | HC7T Vs MS-7T | HC-3T Vs HC-7T | MS-3T Vs MS-7T |
| --- | --- | --- | --- | --- | --- | --- | --- | --- | --- | --- | --- | --- | --- | --- | --- | --- | --- | --- | --- | --- | --- |
|  | mean ± SD | mean ± SD | mean ± SD | mean ± SD | F_83_ | pFDR | pFDR | pFDR | pFDR | pFDR |  | mean ± SD | mean ± SD | mean ± SD | mean ± SD | F_83_ | pFDR | pFDR | pFDR | pFDR | pFDR |
|  | **Left hemisphere** | | | | | | | | | |  | **Right hemisphere** | | | | | | | | | |
| Caudalmiddlefrontal | 21.85 ± 1.46 | 50.47 ± 3.92 | 30.1 ± 2.98 | 51.27 ± 8.66 | 353.66 | **5.2E-30** | **2.9E-08** | 0.94 | **2.6E-07** | **2.6E-07** |  | 22.04 ± 1.32 | 46.83 ± 3.55 | 30.21 ± 3.8 | 48.79 ± 10.4 | 194.55 | **5.2E-25** | **9.3E-09** | 0.23 | **7.3E-09** | **3.8E-09** |
| Lateralorbitofrontal | 22.49 ± 1.37 | 55.4 ± 3.67 | 31.04 ± 1.94 | 57.49 ± 5.42 | 827.30 | **1.7E-36** | **2.6E-07** | 0.57 | **1.3E-07** | **1.3E-07** |  | 22.18 ± 1.43 | 52.15 ± 2 | 29.63 ± 1.93 | 56.69 ± 7.87 | 299.57 | **1.2E-28** | **2.5E-07** | 0.99 | **7.1E-09** | **8.5E-09** |
| Medialorbitofrontal | 22.31 ± 1.73 | 52.63 ± 1.63 | 30.45 ± 1.23 | 53.79 ± 5.35 | 414.18 | **3.2E-31** | **9.4E-09** | 0.82 | **8.5E-08** | **8.5E-08** |  | 23.28 ± 1.22 | 55.93 ± 3.81 | 30.72 ± 1.99 | 55.96 ± 7.81 | 237.87 | **1.0E-26** | **7.9E-06** | 0.3 | **6.9E-09** | **4.4E-09** |
| Paracentral | 16.93 ± 1.36 | 38.47 ± 5.66 | 23.78 ± 2.99 | 37.91 ± 6.23 | 255.63 | **2.5E-27** | **1.8E-08** | 0.97 | **6.4E-08** | **3.9E-09** |  | 16.86 ± 1.49 | 36.25 ± 4.84 | 24.46 ± 2.72 | 39.5 ± 5.71 | 259.20 | **1.9E-27** | **4.3E-08** | 0.37 | **6.7E-09** | **4.2E-09** |
| Parsopercularis | 22.27 ± 1.32 | 55.69 ± 4.17 | 29.27 ± 2.54 | 56.11 ± 6.66 | 720.81 | **1.3E-35** | **1.3E-07** | 0.98 | **5.1E-08** | **6.4E-08** |  | 22.55 ± 1.15 | 52.27 ± 2.29 | 29.54 ± 3.23 | 55.87 ± 8.18 | 388.25 | **1.0E-30** | **1.0E-08** | 0.49 | **6.6E-09** | **8.3E-09** |
| Parsorbitalis | 22.95 ± 1.67 | 56.56 ± 2.39 | 31.24 ± 2.72 | 58.73 ± 7.52 | 550.03 | **1.6E-33** | **5.1E-08** | 0.78 | **4.3E-08** | **5.1E-08** |  | 22.73 ± 1.43 | 55.73 ± 2.32 | 31 ± 3.43 | 59.52 ± 8.76 | 329.22 | **1.9E-29** | **2.4E-08** | 0.15 | **6.4E-09** | **8.0E-09** |
| Parstriangularis | 22.37 ± 1.24 | 55.06 ± 3.66 | 29.75 ± 2.32 | 57.12 ± 7.76 | 549.92 | **1.6E-33** | **2.0E-08** | 0.81 | **3.7E-08** | **4.3E-08** |  | 22.69 ± 1.24 | 51.11 ± 1.99 | 30.46 ± 3.04 | 55.78 ± 9.11 | 376.29 | **1.7E-30** | **1.3E-08** | 0.99 | **6.3E-09** | **7.8E-09** |
| Precentral | 17.35 ± 1.23 | 41.39 ± 4.43 | 23.69 ± 2.5 | 40.08 ± 6.83 | 337.90 | **1.3E-29** | **1.6E-08** | 0.86 | **3.2E-08** | **4.5E-09** |  | 17.31 ± 1.32 | 39.07 ± 3.68 | 23.62 ± 2.61 | 39.02 ± 7.01 | 273.79 | **6.8E-28** | **1.1E-08** | 0.07 | **6.1E-09** | **4.3E-09** |
| Rostralmiddlefrontal | 23.32 ± 1.2 | 54.22 ± 3.09 | 31.48 ± 2.4 | 57.19 ± 7.21 | 495.41 | **1.1E-32** | **2.6E-08** | 0.44 | **2.8E-08** | **3.7E-08** |  | 23.9 ± 1.2 | 51.45 ± 2.22 | 31.32 ± 2.4 | 57.65 ± 9.01 | 285.60 | **3.2E-28** | **4.8E-08** | 0.28 | **6.0E-09** | **7.5E-09** |
| Superiorfrontal | 23.1 ± 1.37 | 49.68 ± 3.91 | 32.1 ± 3.23 | 53.43 ± 8.48 | 331.23 | **1.7E-29** | **8.5E-08** | 0.32 | **2.6E-08** | **3.2E-08** |  | 23.67 ± 1.36 | 49.69 ± 3.03 | 32.06 ± 2.77 | 53.78 ± 8.21 | 297.76 | **1.4E-28** | **1.3E-08** | 0.75 | **5.8E-09** | **7.3E-09** |
| Frontalpole | 23.74 ± 1.88 | 54.96 ± 3.71 | 31.63 ± 3.55 | 59.18 ± 6.93 | 276.77 | **5.5E-28** | **4.0E-07** | 0.36 | **2.3E-08** | **2.8E-08** |  | 23.07 ± 1.74 | 53.83 ± 3.03 | 30.12 ± 3.27 | 57.1 ± 8.19 | 226.80 | **2.6E-26** | **1.2E-05** | **0.0022** | **5.7E-09** | **7.1E-09** |
| Caudalanterior-cingulate | 26.91 ± 1.88 | 53.39 ± 4.9 | 37.61 ± 2.04 | 60.96 ± 5.35 | 267.92 | **1.0E-27** | **9.5E-09** | **0.0096** | **2.1E-08** | **2.6E-08** |  | 26.65 ± 1.65 | 53.39 ± 2.8 | 33.83 ± 2.08 | 61.29 ± 5.24 | 357.30 | **4.4E-30** | **3.4E-07** | 0.76 | **5.6E-09** | **6.9E-09** |
| Isthmuscingulate | 20.72 ± 1.18 | 40.9 ± 4.49 | 25.91 ± 1.03 | 41.66 ± 5.76 | 154.44 | **4.9E-23** | **3.4E-05** | 0.93 | **2.0E-08** | **3.9E-09** |  | 19.93 ± 1.36 | 35.26 ± 4.09 | 24.39 ± 1.83 | 37.91 ± 7.47 | 71.20 | **1.0E-16** | **0.0024** | 0.14 | **3.8E-09** | **6.8E-09** |
| Posteriorcingulate | 23.42 ± 1.56 | 46.27 ± 2.9 | 30.24 ± 1.78 | 49.12 ± 5.49 | 257.56 | **2.2E-27** | **3.2E-08** | 0.43 | **1.8E-08** | **2.3E-08** |  | 23.11 ± 1.27 | 44.24 ± 3.28 | 29.11 ± 1.99 | 48.41 ± 5.91 | 239.12 | **9.3E-27** | **2.2E-07** | 0.15 | **5.5E-09** | **6.7E-09** |
| Rostralanterior-cingulate | 24.84 ± 1.62 | 57 ± 6.18 | 31.9 ± 2.61 | 60.04 ± 7.75 | 255.09 | **2.5E-27** | **3.1E-06** | 0.78 | **1.7E-08** | **2.1E-08** |  | 24.93 ± 1.56 | 58.57 ± 2.19 | 32.9 ± 1.08 | 64.03 ± 8.45 | 332.58 | **1.6E-29** | **3.0E-07** | 0.82 | **5.3E-09** | **6.6E-09** |
| Inferiorparietal | 22.68 ± 1.37 | 45.64 ± 4.35 | 30 ± 1.24 | 48.7 ± 6.13 | 314.27 | **4.9E-29** | **1.4E-08** | 0.32 | **1.6E-08** | **2.0E-08** |  | 20.95 ± 1.4 | 51.22 ± 2.63 | 27.85 ± 2.15 | 52.8 ± 6.69 | 471.19 | **2.7E-32** | **9.1E-09** | 0.63 | **5.2E-09** | **6.4E-09** |
| Postcentral | 17.97 ± 1.28 | 42.11 ± 4.31 | 24.67 ± 1.49 | 42.94 ± 6.41 | 386.21 | **1.1E-30** | **2.3E-08** | 0.93 | **1.5E-08** | **1.8E-08** |  | 17.35 ± 1.2 | 40.06 ± 2.78 | 23.85 ± 1.78 | 42.42 ± 6.8 | 346.27 | **7.8E-30** | **1.4E-08** | 0.12 | **5.1E-09** | **6.3E-09** |
| Precuneus | 21.27 ± 1.41 | 46.78 ± 3.98 | 27.78 ± 1.62 | 48.46 ± 5.76 | 396.98 | **6.7E-31** | **1.1E-08** | 0.87 | **1.4E-08** | **1.7E-08** |  | 20.37 ± 1.24 | 42.76 ± 3.32 | 26.75 ± 2.34 | 47.03 ± 6.46 | 304.22 | **9.3E-29** | **1.1E-08** | 0.36 | **5.0E-09** | **6.1E-09** |
| Superiorparietal | 20.74 ± 1.28 | 42.36 ± 3.77 | 27.96 ± 1.86 | 44.96 ± 7.34 | 225.74 | **2.8E-26** | **8.9E-09** | 0.65 | **1.3E-08** | **4.1E-09** |  | 19.33 ± 1.17 | 42.86 ± 2.28 | 26.43 ± 2.25 | 45.66 ± 6.12 | 384.26 | **1.2E-30** | **2.1E-08** | 0.82 | **4.9E-09** | **6.0E-09** |
| Supramarginal | 22.76 ± 1.33 | 50.44 ± 4.58 | 30.14 ± 1.94 | 52.28 ± 6.66 | 403.42 | **5.0E-31** | **1.5E-08** | 0.86 | **1.3E-08** | **1.6E-08** |  | 21.72 ± 1.34 | 51.03 ± 1.38 | 28.19 ± 2.51 | 52.71 ± 7.39 | 383.79 | **1.2E-30** | **2.1E-08** | 0.93 | **4.8E-09** | **5.8E-09** |
| Bankssts | 24.08 ± 1.64 | 55.73 ± 6.65 | 31.6 ± 2.13 | 55.59 ± 6.82 | 360.32 | **3.9E-30** | **9.1E-09** | 0.99 | **1.2E-08** | **1.5E-08** |  | 22.85 ± 1.64 | 57.69 ± 2.93 | 29.69 ± 2.31 | 56.95 ± 6.77 | 478.41 | **2.1E-32** | **1.6E-08** | 0.93 | **4.7E-09** | **5.7E-09** |
| Entorhinal | 20.9 ± 1.92 | 53.69 ± 7.69 | 25.45 ± 2.51 | 53.86 ± 5.11 | 224.02 | **3.2E-26** | **0.0068** | 0.99 | **1.2E-08** | **1.4E-08** |  | 20.84 ± 2.21 | 56.2 ± 3.43 | 25.61 ± 3.17 | 55.57 ± 4.85 | 336.87 | **1.3E-29** | **0.0016** | 0.93 | **4.7E-09** | **5.6E-09** |
| Fusiform | 21.81 ± 1.2 | 55.98 ± 3.77 | 28.19 ± 1.46 | 57.98 ± 5.06 | 676.52 | **3.3E-35** | **1.1E-08** | 0.71 | **1.1E-08** | **1.3E-08** |  | 20.42 ± 1.15 | 55.83 ± 2.44 | 26.56 ± 1.57 | 56.35 ± 4.39 | 914.77 | **4.0E-37** | **9.8E-09** | 0.85 | **4.6E-09** | **5.5E-09** |
| Inferiortemporal | 23.81 ± 1.39 | 54.07 ± 3 | 31.23 ± 1.72 | 59.67 ± 5.1 | 539.48 | **2.1E-33** | **9.2E-09** | **0.013** | **1.1E-08** | **1.3E-08** |  | 22.18 ± 1.22 | 55.54 ± 3.54 | 29.18 ± 1.9 | 56.98 ± 6.63 | 451.88 | **5.8E-32** | **3.5E-08** | 0.59 | **4.5E-09** | **5.3E-09** |
| Middletemporal | 24.28 ± 1.39 | 55.18 ± 3.99 | 30.9 ± 1.65 | 57.3 ± 5.84 | 634.60 | **1.0E-34** | **1.7E-08** | 0.61 | **1.0E-08** | **1.2E-08** |  | 23.77 ± 1.33 | 58.94 ± 1.28 | 30.57 ± 1.8 | 61.34 ± 5.52 | 770.72 | **5.0E-36** | **1.1E-08** | 0.1 | **4.4E-09** | **5.2E-09** |
| Parahippocampal | 22.06 ± 1.84 | 47.31 ± 7.27 | 26.6 ± 1.68 | 53.03 ± 4.12 | 207.28 | **1.5E-25** | **0.0084** | 0.09 | **9.9E-09** | **1.2E-08** |  | 21.42 ± 1.82 | 48.2 ± 3.61 | 25.95 ± 2.42 | 53.26 ± 5.16 | 285.02 | **3.2E-28** | **9.8E-04** | 0.71 | **4.3E-09** | **5.1E-09** |
| Superiortemporal | 22.39 ± 1.14 | 55.52 ± 4.84 | 29.7 ± 1.45 | 55.8 ± 5.38 | 653.71 | **6.0E-35** | **3.2E-08** | 0.98 | **9.5E-09** | **1.1E-08** |  | 22.03 ± 1.26 | 56.02 ± 1.65 | 29.04 ± 2.12 | 58.25 ± 6.09 | 693.35 | **2.3E-35** | **1.1E-08** | 0.98 | **4.3E-09** | **5.0E-09** |
| Temporalpole | 23.37 ± 2.19 | 59.91 ± 5.91 | 30.53 ± 2.66 | 60.92 ± 5.94 | 284.44 | **3.3E-28** | **3.6E-05** | 0.93 | **9.2E-09** | **1.1E-08** |  | 22.49 ± 1.69 | 62.4 ± 4.51 | 30.97 ± 2.15 | 62.83 ± 9.85 | 217.24 | **5.9E-26** | **1.1E-04** | 0.86 | **4.2E-09** | **4.3E-09** |
| Transversetemporal | 15.24 ± 0.97 | 34.73 ± 4.81 | 20.92 ± 2.64 | 37.08 ± 7.34 | 205.51 | **1.8E-25** | **2.6E-08** | 0.76 | **8.8E-09** | **4.0E-09** |  | 14.37 ± 1.17 | 35.14 ± 3.7 | 19.63 ± 2.27 | 37.43 ± 8.05 | 186.35 | **1.2E-24** | **1.2E-06** | 0.1 | **4.1E-09** | **4.1E-09** |
| Insula | 18.84 ± 1.23 | 45.39 ± 5.42 | 25.41 ± 1.34 | 49.18 ± 5.59 | 424.50 | **2.0E-31** | **9.4E-09** | 0.13 | **8.5E-09** | **1.0E-08** |  | 18.3 ± 1.29 | 47.41 ± 3.26 | 26.15 ± 2.03 | 53.5 ± 8.42 | 268.56 | **9.8E-28** | **3.0E-07** | **0.013** | **4.1E-09** | **4.9E-09** |
| Cuneus | 17.02 ± 1.06 | 40.79 ± 3.65 | 23.44 ± 0.82 | 43.19 ± 4.11 | 457.30 | **4.7E-32** | **1.2E-08** | 0.34 | **8.3E-09** | **9.9E-09** |  | 16.25 ± 1.05 | 37.76 ± 2.49 | 22.73 ± 1.25 | 42.66 ± 4.61 | 404.45 | **5.0E-31** | **1.0E-08** | 0.33 | **4.0E-09** | **4.8E-09** |
| Lateraloccipital | 19.41 ± 1.18 | 43.02 ± 4.27 | 25.85 ± 1.13 | 47.19 ± 4.75 | 539.51 | **2.1E-33** | **6.4E-08** | **0.022** | **8.0E-09** | **9.5E-09** |  | 18.09 ± 1.04 | 46.97 ± 2.59 | 24.5 ± 1.81 | 49.64 ± 5.5 | 717.84 | **1.3E-35** | **3.7E-08** | 0.07 | **3.9E-09** | **4.7E-09** |
| Lingual | 16.85 ± 1.05 | 42.47 ± 5.19 | 22.6 ± 0.61 | 45.36 ± 4.57 | 490.11 | **1.3E-32** | **9.0E-09** | 0.23 | **7.8E-09** | **9.2E-09** |  | 16.07 ± 0.84 | 39.82 ± 4.47 | 21.87 ± 0.78 | 44.08 ± 5.02 | 366.08 | **2.9E-30** | **8.0E-08** | 0.15 | **3.9E-09** | **4.7E-09** |
| Pericalcarine | 15.08 ± 1.36 | 38.31 ± 5.23 | 20.4 ± 0.6 | 39.9 ± 4.38 | 356.88 | **4.4E-30** | **2.3E-08** | 0.87 | **7.5E-09** | **8.8E-09** |  | 13.87 ± 1.23 | 35.11 ± 3.95 | 19.76 ± 0.64 | 38.85 ± 5.19 | 284.20 | **3.3E-28** | **5.7E-08** | 0.15 | **3.8E-09** | **4.6E-09** |

Mean regional values are presented ± standard deviation (SD). Bold letters indicate significant (p < 0.05, corrected for multiple comparisons using FDR) differences.
